# From fracture to function: return-to-sport after acetabular fractures

**DOI:** 10.1016/j.jor.2025.12.053

**Published:** 2025-12-23

**Authors:** Anna L. Schiltenwolf, Tina Histing, Florian Laux, Markus A. Kueper, Stefan Doebele, Steven C. Herath

**Affiliations:** a Department of Trauma and Reconstructive Surgery, Eberhard Karls University Tuebingen, BG Trauma Center Tuebingen, 72076, Tuebingen, Germany; b Health Care Center and Rehabilitation Clinic Swabian Alb, 72574, Bad Urach, Germany; c Department of Sports Medicine/Sports Orthopedics, University Hospital Tuebingen, Tuebingen, Germany

**Keywords:** Acetabular fracture, Return to sport, Maximum performance, Subjective attitude towards exercise

## Abstract

**Background and aim of the study:**

Acetabular fractures can affect young, athletically active patients. This study aimed to describe the previously unexplored interaction between injury severity, return to sport, and subjective attitude towards sport.

**Material and methods:**

Twenty-two patients with acetabular fracture treated at a Level I Trauma Center between Jan. 2009–Dec. 2020 were retrospectively included. Patients received a postal questionnaire assessing activity level and sporting activity before trauma (PRE) and after trauma (POST). The median follow-up was 145 (IQR 75.5–166) months. Patients also reported whether the injury altered their subjective attitude towards exercise.

**Results:**

Participants were divided into two subgroups according to ISS: ISS <16 = ‘no polytrauma’ (n = 8) and ISS ≥16 = ‘polytrauma’ (n = 14). The mean highest UCLA Activity Score decreased more in the ‘no polytrauma’ subgroup (−39.7 %) than in the ‘polytrauma’ subgroup (−23.4 %). The POST sports break lasted over five times longer in the ‘polytrauma’ subgroup, who more often underwent longer inpatient rehabilitation. In the ‘no polytrauma’ subgroup, 50 % (3/6) of previously active athletes returned to sport, whereas all PRE active patients in the ‘polytrauma’ subgroup returned (10/10). POST sport level was lower in the ‘no polytrauma’ subgroup (0 % club sports (0/8), 37.5 % occasional (3/8); 0 % high-impact (0/8), 37.5 % low-impact (3/8)) compared with the ‘polytrauma’ subgroup (21.4 % club sports (3/14), 50 % occasional (7/14); 35.7 % high-impact (5/14), 35.7 % low-impact (3/8)). A negative change in the subjective attitude towards exercise occurred in 12.5 % (1/8) of ‘no polytrauma’ patients and 42.9 % (6/14) of ‘polytrauma’ patients.

**Conclusion:**

Sporting activity was reduced after acetabular fracture. Although ‘polytrauma’ participants reported more negative shifts in exercise attitude, they demonstrated higher return-to-sport rates compared with ‘non-polytrauma’ participants. Potentially the longer sport interruption and inpatient rehabilitation may facilitate more effective reintegration. Further studies with larger study groups are warranted.

## 1 Introduction

Acetabular fractures show a bimodal age distribution.^1^ In younger patients they are mainly caused by high-speed trauma.^2^ Treatment aims to restore the acetabulum, independence, and quality of life.^3^^,^^4^ Physical activity provides well-documented physical and psychosocial benefits and is influenced by individual attitudes toward sport.^5^, ^6^, ^7^

Activity levels often decline after an acetabular fracture. While 67–81 % return to sport, the radiological quality of reduction is a key predictor of post-trauma sport levels, whereas both-column and posterior wall fractures are associated with lower participation.^8^^,^^9^

The Injury Severity Score (ISS) for isolated acetabular fractures averages 10–16.^10^ Polytrauma patients (ISS ≥16) show significantly reduced EQ-5D scores (ISS <16).^11^

However, no studies have examined how overall injury severity affects return-to-sport or activity levels after acetabular fracture. This study investigated whether injury severity (polytrauma vs. non-polytrauma) affects return-to-sport, post-traumatic activity level, maximum performance, and subjective attitudes toward sporting activity after acetabular fractures. We also assessed whether the painkiller use is modified by the injury and its severity.

### 1.1 Patients

The present study included patients with^1^ acetabular fracture treated at a Level 1 Trauma Center between January 2009 and December 2020. Eligible patients were identified from the pelvic registry,^2^ provided informed consent, and^3^ were of working age (18–65 years) at the time of trauma.

### 1.2 Material und methods

Data on postal dispatch, socio-demographics, epidemiological classification, injury type, and accident date were obtained from the Level 1 Trauma Center pelvic registry. Acetabular fractures were classified according to Letournel, individual injury severity by the AIS, and overall severity by the ISS.^12^

A rating system developed at the University of California, Los Angeles (UCLA Activity Score) was used to define the highest activity level pre-trauma (PRE) and post-trauma (POST). The scale ranged from 1 = *completely inactive* to 10 = *vigorous physical work or contact sports*.^13^ The questionnaire assessed sport aspects, including activity level, training frequency and duration, impact level, painkiller use during sport, and POST sport breaks and changes in sport. The term “painkiller” was not further specified and was interpreted individually by participants.^14^

Finally, participants reported how their subjective attitude toward exercise had changed due to the trauma.

### 1.3 Data collection and analysis

The study population was contacted by post in October 2021 with an information letter, consent form, and questionnaire containing retrospective patient-reported outcome measures (PROMs). Completed questionnaire and consent form were returned by mail, missing data were clarified by telephone. All consenting patients were evaluated pseudonymously. No physical examinations, imaging, or personal contact occurred.

Data analysis was purely descriptive, as the cohort comprised only 22 patients and subgroup sizes were too small for adequately powered statistical testing.

The study was reviewed by the local ethics committee (project no. 760/2021BO2), which raised no objections.

## 2 Results

### 2.1 Participants

A total of 544 patients were recorded in the pelvic registry during the study period (396 pelvic ring-, 149 acetabular fractures). Addresses were available for 290 patients, including 56 eligible acetabular fracture cases.

**Fig. 1:**
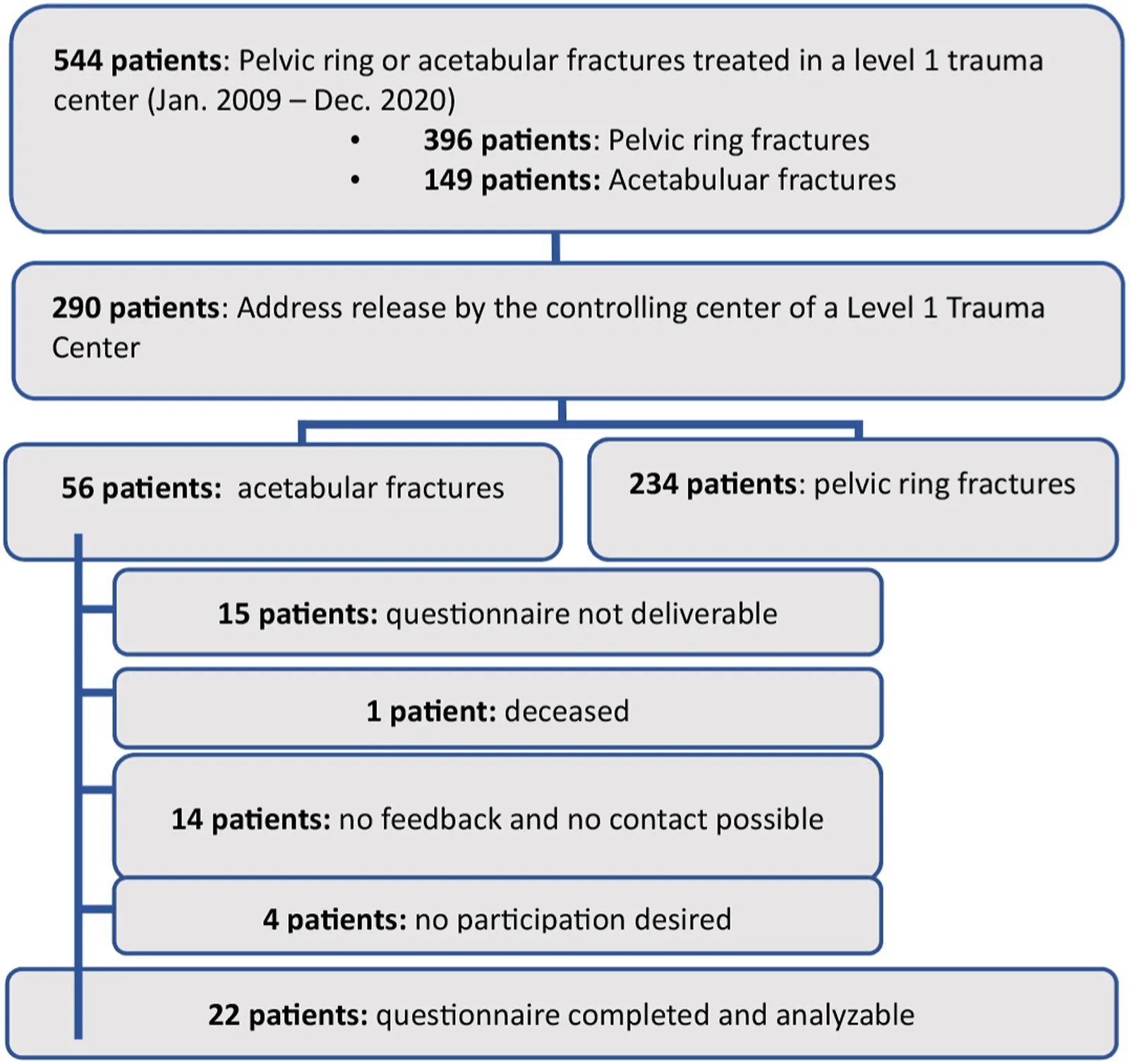
Flowchart of participants.

Fifteen patients could not be contacted, and one patient was deceased, leaving 40 questionnaires successfully mailed. Fourteen patients did not respond despite repeated contact attempts, and four declined participations. Thus, 22 fully completed questionnaires were available for evaluation. (response rate 22/40 = 55 %) (Fig. 1).

We included 7 women (31.8 %) and 15 men (68.2 %) with a median age at the time of trauma of 45 (IQR 33.5–54.75) years. The median time between trauma and sending the questionnaire was 145.0 (IQR 75.5–166) months.

### 2.2 Injury and rehabilitation

There were 15 elementary fractures (68.2 %) and 7 combined patterns (31.8 %) according to Letournel. Based on the AIS, 16 patients (72.7 %) had an AIS 3 injury; 4 of them (18.2 %) sustained an additional Tile B pelvic ring injury. Six patients (27.3 %) had an AIS 4 injury with a concomitant Tile C pelvic ring fracture. In total, 10/22 patients (45.5 %) had an associated pelvic ring injury.

According to the ISS, 14/22 (63.6 %) were classified as polytrauma and 8/22 (36.4 %) as non-polytrauma. The mean ISS of the entire cohort was 23.4 ± 15.0 (range 9–59). Mean ISS was 9.8 ± 1.5 (range 9–13) in the non-polytrauma subgroup and 31.2 ± 13.5 (range 17–59) in the polytrauma subgroup.

Polytrauma patients more frequently underwent inpatient rehabilitation, while non-polytrauma patients more often participated in outpatient programs. Subgroup details are shown in Table 1.

### 2.3 Maximum performance

Overall, the highest UCAL Activity Score declined markedly after acetabular fractures. Both subgroups demonstrated a reduction, which was more pronounced in patients without polytrauma. Detailed results are presented in Table 2, Table 3.

### 2.4 Return to sport

**Fig. 2:**
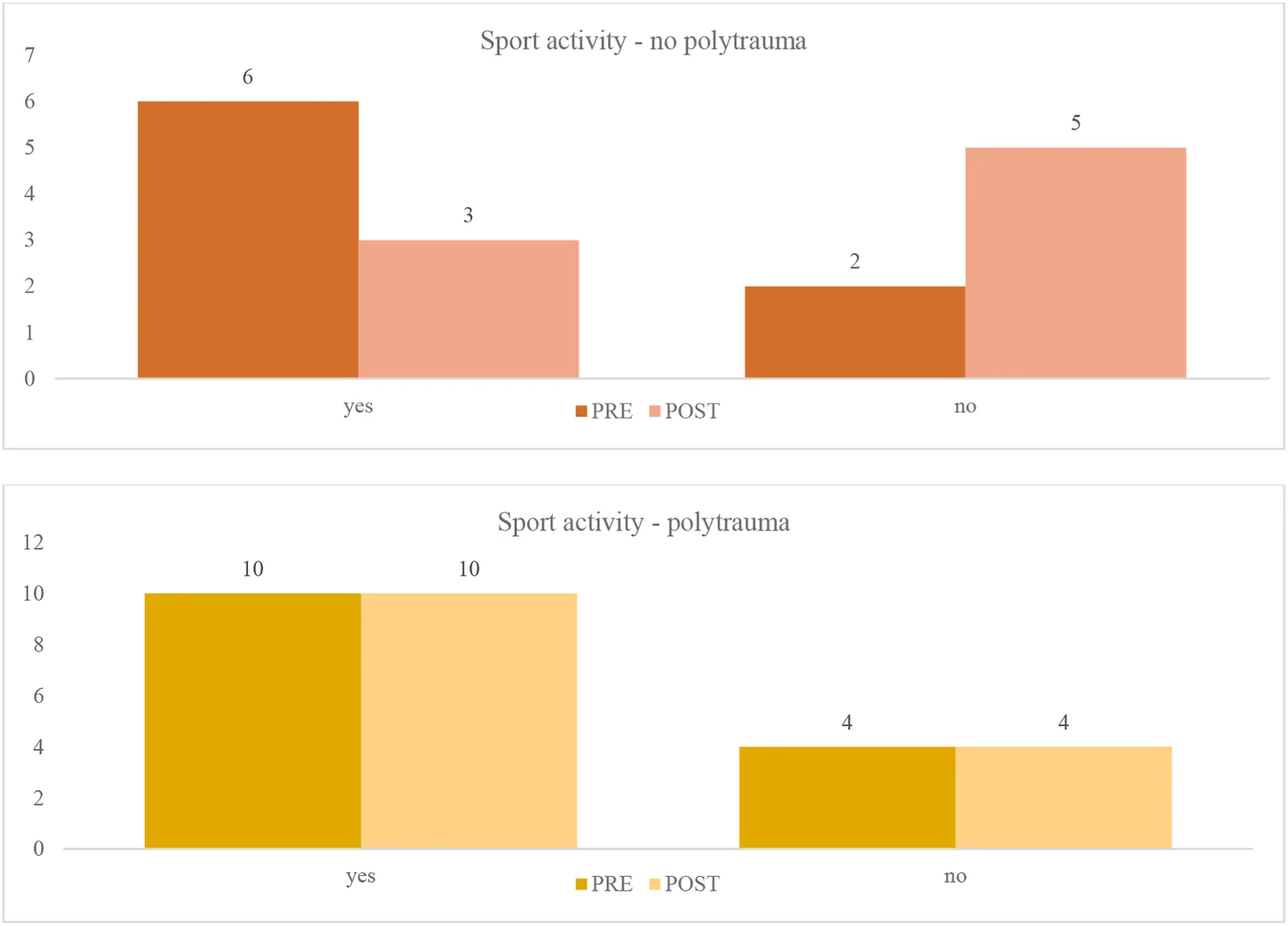
shows the **sport activity** (y-axis: number of patients; x-axis: answer in questionnaire): Most patients were engaged in sports prior to the fracture. Among those previously active, return-to-sport rates were lower in the non-polytrauma subgroup, whereas all polytrauma patients resumed sport POST.

Of the patients with pre-trauma sporting activity, 81.3 % (13/16) returned to sport, while 18.8 % (3/16) did not. Return rates differed between subgroups: 50 % (3/6) in the non-polytrauma group versus 100 % (10/10) in the polytrauma group (Fig. 2).

The POST sports break was markedly longer in the polytrauma subgroup (median 459 days, IQR 135.5–730) than in the non-polytrauma subgroup (median 90 days, IQR 0). Sporting activity levels declined in both groups (Table 4).

Before injury, polytrauma patients more often participated in club sports, whereas non-polytrauma patients mainly performed casual activities. POST, both groups reduced activity level and intensity, with a more marked decline in the non-polytrauma subgroup. Non-polytrauma patients trained less frequently but longer PRE, and more frequently but shorter POST, resulting in a major net reduction. Polytrauma patients showed only moderate decreases, and both groups largely discontinued high-impact sports. (Table 4).

Use of pain medication during activity increased in both subgroups. Most non-polytrauma patients who had trained without analgesics required pain medication POST, whereas polytrauma patients—despite increased analgesic use—more often continued exercising without pain medication.

### 2.5 Subjective attitude towards sporting activity

**Fig. 3:**
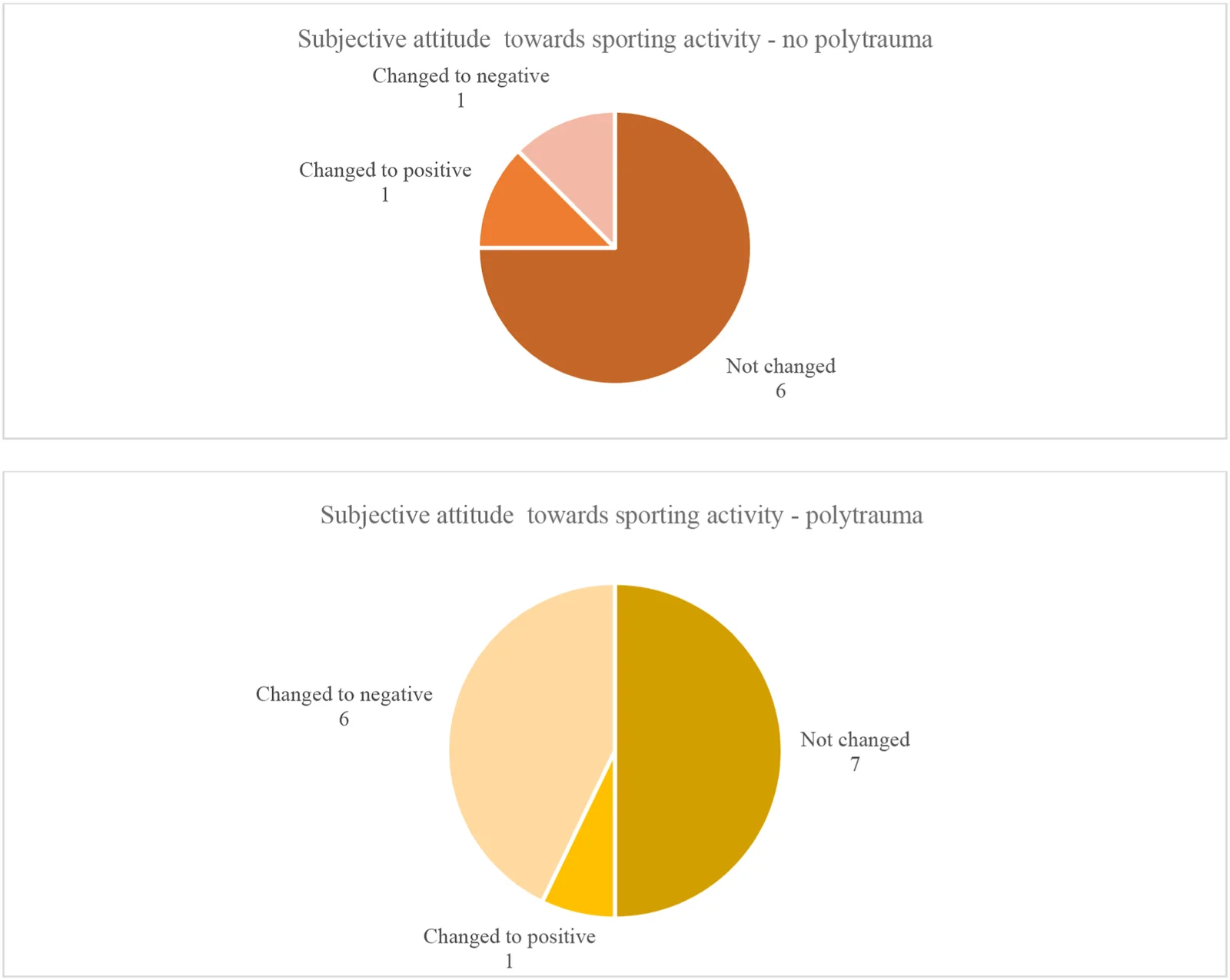
shows change the **subjective attitude towards sporting activity:** Most patients in the ‘no polytrauma’ subgroup reported no change, while in the polytrauma subgroup negative shifts in attitude were more common.

Overall, most patients (59 %; 13/22) reported no change in their general attitude towards sporting activity. In the ‘non-polytrauma’ subgroup, attitudes towards sporting activity were largely stable, with only few positive or negative shifts. In contrast, half of the patients in the ‘polytrauma’ subgroup reported no change, while negative shifts in attitude were more frequent than positive ones (Fig. 3).

## 3 Discussion

### 3.1 Return to sport and maximum performance

Almost 20 % of the cohort did not resume sporting activity after an acetabular fracture.

The subgroup analysis shows that patients with polytrauma are more likely to resume sporting activity than those without polytrauma. The contrast between the subgroups was striking: patients without polytrauma returned to sport earlier, but fewer of them resumed sporting activity and at a lower activity level. Rehabilitation was undertaken less frequently in this subgroup. In contrast, all patients in the ‘polytrauma’ subgroup eventually returned to sport after a longer interruption, underwent more frequently inpatient rehabilitation and reached higher activity level compared to the ‘no polytrauma’ subgroup. In line with this, Yabroudi et al. (2021) demonstrated that a longer rehabilitation period (≥4 months) was positively correlated with return to sport after anterior cruciate ligament surgery, which could help explain the findings of the present study.^15^

Patients in the polytrauma subgroup showed higher activity levels and more frequent sporting activity both before and after trauma, suggesting that sport played a more prominent role in their daily lives.

Our study is the first to characterize pre- and post-traumatic physical activity in patients with acetabular fractures.

### 3.2 Painkiller consumption on return to sport

Following acetabular fractures, painkiller use during sporting activity increased, particularly in the ‘polytrauma’ subgroup. This corresponds with Monteleone et al. (2023), who identified postoperative pain as the major limitation after acetabular fractures.^16^

### 3.3 Subjective attitude towards sporting activity

Most patients reported no change in their subjective attitude toward sporting activity, although a relevant share described a negative shift. This was more frequent and predominantly negative in the polytrauma subgroup, while attitudes in the non-polytrauma subgroup remained largely stable. To our knowledge, this is the first study to examine attitude changes toward sport after acetabular fracture.

Physical activity is associated with numerous benefits, including reduced depression, pain, blood pressure, and cognitive decline, as well as improved self-esteem and quality of life.^5^^,^^17^^,^^18^ Conversely, depression can reduce activity levels.^19^ Moreover, subjective sport perception strongly influences participation, with positive attitudes promoting and negative attitudes limiting performance.^7^

A negative attitude toward exercise may contribute to lower activity levels and adverse biopsychosocial outcomes. In this study, a notable proportion of patients showed a negative shift in attitude; however, this did not parallel the UCLA Activity Score. Although activity decreased more in the non-polytrauma subgroup, negative attitude changes were more common in polytrauma patients. Further factors such as motivation, reasons for attitude changes, and subjective experience during activity should be explored, particularly as sport played a greater role pre- and post-trauma in the polytrauma subgroup.

## 4 Limitations

The main limitation of this study is the small sample size. Selection and nonresponse bias are likely, as only a subset of contacted patients participated. A larger cohort is required for meaningful comparisons and adequately powered analyses.

Including patients with concomitant pelvic ring injuries prevents isolated interpretation of acetabular fracture effects, and no causal conclusions can be drawn regarding changes in work or sports resilience or the role of polytrauma. The retrospective design introduces recall and reporting bias. It also limits verification of the accuracy of patient-reported data.

The term “painkillers” was not further defined, leaving medication type and dosage to individual interpretation. Finally, due to the descriptive nature of the study, additional confounding factors (e.g., age, relocation, social environment) that may have influenced occupational or sporting activity independently of the trauma could not be controlled for.

## 5 Conclusion

The sporting activity and physical performance declined in the overall cohort after acetabular fracture. Although participants with polytrauma reported more negative changes in their attitude towards exercise than those without polytrauma, they achieved higher return-to-sport rates and better physical performance. In the ‘polytrauma’ subgroup, POST sport interruption was more than five times longer, and they more frequently underwent prolonged inpatient rehabilitation. This extended recovery period may have faciliated more effective reintegration into sporting activity. Further studies with larger cohorts are necessary for meaningful comparisons and adequately powered analyses.

## Informed consent

All patients provided informed consent for participation and data collection/storage.

## Ethical approval

The study was advised in accordance with the ethical standards laid down in an appropriate version of the 1964 Declaration of Helsinki. The study was advised by the ethics committee of the medical faculty under the project number 760/2021BO2. There were no objections to conducting the study.

## Authors contributions

Anna L. Schiltenwolf, Markus A. Kueper and Florian Laux conceptualized the study and oversaw project administration. They also designed the data collection process and conducted data analysis. Anna L. Schiltenwolf drafted the initial manuscript. Steven C. Herath, Stefan Doebele, and Tina Histing critically reviewed and approved the final version of the manuscript. All authors read and approved the final version.

## Funding

We acknowledge support from the Open Access Publication Fund of the University of Tübingen.

## Conflict of interest

All authors declare that they have no competing interests.

## Figures and Tables

**Table 1:** Characteristics of the subgroups

	No polytrauma (ISS<16)	Polytrauma (ISS≥16)
Age in years		
Median	49.5 (IQR 37.5–55.5)	43 (IQR 25–51.75)
Men: women	7 : 1	8 : 6
Body height in cm		
Median	178 (IQR 175.5–183.75)	171 (IQR 166.75–180.5)
Body weight in kg		
Median	82 (IQR 175.5–183.75)	78 (IQR 72–88)
AIS-Score		
3	8/8 (100 %)	8/14 (57,1 %)
4	–	6/14 (42,9 %)
Additional pelvic ring fracture		
Tile B	1/8 (12,5 %)	3/14 (21,4 %)
Tile C	–	6/14 (42,9 %)
Letournel/Judet		
anterior wall fracture	2/8 (25 %)	–
anterior column fracture	4/8 (50 %)	2/14 (14,3 %)
posterior column fracture	–	1/14 (7,1 %)
transverse fracture	–	6/14 (42,8 %)
transverse + posterior wall fracture	1/8 (12,5 %)	3/14 (21,4 %)
both column fracture	1/8 (12,5 %)	2/14 (14,3 %)
Treatment pelvis		
conservative	3/8 (37,5 %)	1/14 (7 %)
one operation	2/8 (25 %)	3/14 (14,3)
multiple operations	3/8 (37,5 %)	10/14 (57,1 %)
Rehabilitation		
yes	5/8 (62,5 %)	13/14 (92,9 %)
no	3/8 (37,5 %)	1/14 (7,1 %)
Duration outpatient rehab in days		
Median (IQR)	60 (60–70)	21 (19.25–38.25)
Duration inpatient rehab in days		
Median (IQR)	35 (31.5–41.25)	61.5 (33.5–89.25)

**Table 2:** UCLA Activity Score; No polytrauma

No polytrauma	PRE (n)	POST (n)	Difference (n)
**Regularly participates in impact sports**	1/8 (12,5 %)	0	−1 (−12.5 %)
**Sometime participates in impact sports**	2/8 (25 %)	0	−2 (−25 %)
**Regularly participates in very active events**	0	0	0
**Regularly participates in active events**	2/8 (25 %)	1/8 (12,5 %)	−1 (−12,5 %)
**Regularly participates in moderate activities**	2/8 (25 %)	2/8 (25 %)	0
**Sometime participates in moderate activities**	0	0	0
**Regularly participates in mild activities**	1/8 (12,5 %)	2/8 (25 %)	+1 (+12,5 %)
**Sometime participates in mild activities**	0	2/8 (25 %)	+2 (+25 %)
**Mostly inactive**	0	1/8 (12,5 %)	+1 (+12,5 %)
**Wholly inactive**	0	0	0

**Table 3:** UCLA Activity Score; Polytrauma

Polytrauma	PRE (n)	POST (n)	Difference (n)
**Regularly participates in impact sports**	2/14 (14,3 %)	0	−2 (−14,3 %)
**Sometime participates in impact sports**	4/14 (28,6 %)	3/14 (21,4 %)	−1 (−7,2 %)
**Regularly participates in very active events**	1/14 (7,1 %)	2/14 (14,3 %)	+2 (+7,2 %)
**Regularly participates in active events**	4/14 (28,6 %)	2/14 (14,3 %)	−2 (−14,3 %)
**Regularly participates in moderate activities**	2/14 (14,3 %)	2/14 (14,3 %)	0
**Sometime participates in moderate activities**	0	0	0
**Regularly participates in mild activities**	1/14 (7,1 %)	2/14 (14,3 %)	+1 (+7,2 %)
**Sometime participates in mild activities**	0	1/14 (7,1 %)	+1 (+7,1 %)
**Mostly inactive**	0	1/14 (7,1 %)	+1 (+7,1 %)
**Wholly inactive**	0	1/14 (7,1 %)	+1 (+7,1 %)

**Table 4:** Return to sport

	No polytrauma		Polytrauma	
	PRE	POST	PRE	POST
POST sport break in days				
Median	90 (IQR 0)		459 (IQR 135.5–730)	
Level of sport activity				
Professional	–	–	–	–
Club sport	–	–	4/14 (28,6 %)	3/14 (21,4 %)
Casual sport	6/8 (75 %)	3/8 (37,5 %)	6/14 (42,9 %)	7/14 (50 %)
No sport	2/8 (25 %)	5/8 (62,5 %)	4/14 (28,6 %)	4/14 (28,6 %)
Number of different kind of sports				
Mean ± SD	3 ± 0,9	1,5 ± 0,7	2,1 ± 0,7	2,2 ± 1,2
Sport unit per week				
Mean ± SD	2 ± 0,9	2,5 ± 2,1	3,6 ± 1,1	3,2 ± 1,1
Duration per sport unit in minutes				
Mean ± SD	115 ± 79,2	25 ± 7,1	72,5 ± 30	51,1 ± 26,9
POST change of sport				
yes	2/8 (25 %)		5/14 (35,7 %)	
no	1/8 (12,5 %)		5/14 (35,7 %)	
Sport Impact				
Low- Impact	2/8 (25 %)	3/8 (37,5 %)	2/14 (14,3 %)	5/14 (35,7 %)
High-Impact	4/8 (50 %)	–	8/14 (57,1 %)	5/14 (35,7 %)
Painkiller consumption during sport				
Occasionally	–	2/8 (25 %)	–	3/14 (21,4 %)
Regularly	–	–	–	1/14 (7,1 %)
No	6/8 (75 %)	1/8 (12,5 %)	10/14 (71,4 %)	6/14 (42,9 %)

## Abbreviations

EQ-5D: European Quality of Life 5 Dimensions 5 Level
ISS: Injury Severity Score
M: Mean
MD: Median
PRE: pre-trauma
POST: post-trauma
RTS: Return-to-sport
UCLA: University of California, Los Angeles
